# Viral kinetics among persons living with HIV (PLWH) on Dolutegravir-based antiretroviral Regimen: A retrospective and prospective analysis from selected HIV clinics in Ghana

**DOI:** 10.1371/journal.pone.0324360

**Published:** 2025-05-20

**Authors:** Samuel Badu Nyarko, Joel Adu Twum, Aikins Sarpong Obeng, Helena Dede Djangba, Oksana Ryabinina, Foster Kyei, George Boateng Kyei, Nicholas Ekow Thomford

**Affiliations:** 1 Pharmacogenomics and Genomic Medicine Group, School of Medical Sciences, College of Health and Allied Sciences, University of Cape Coast, Cape Coast, Ghana; 2 Department of Molecular Biology and Biotechnology, School of Biological Sciences, College of Agriculture and Natural Sciences, University of Cape Coast, Cape Coast, Ghana; 3 Department of Medical Biochemistry, School of Medical Sciences, College of Health and Allied Sciences, University of Cape Coast, Cape Coast, Ghana; 4 Department of Chemical Pathology, School of Medical Sciences, College of Health and Allied Sciences, University of Cape Coast, Cape Coast, Ghana; 5 Department of Virology, Noguchi Memorial Institute for Medical Research, University of Ghana, Legon, Ghana; 6 Department of Medicine, Washington University School of Medicine, St. Louis, Missouri, United States of America; 7 Medical and Scientific Research Centre, University of Ghana Medical Centre, Accra, Ghana; 8 Department of Pathology, Faculty of Health Sciences, University of Cape Town, Cape Town, South Africa; University of Ghana, GHANA

## Abstract

**Background:**

Dolutegravir (DTG)-based antiretroviral therapy has demonstrated superior efficacy, tolerability, and durability when compared to other HIV treatment regimens. However, monitoring viral kinetics is critical for determining treatment efficacy and making sound judgments. The purpose of this study was to assess viral kinetics in people living with HIV (PLWH) on DTG-based ART and identify characteristics related to virologic response in the Cape Coast Metropolis, Ghana.

**Methods:**

Among people living with HIV (PLWH) attending HIV clinics between January 2020 and December 2023, a prospective and retrospective analysis of viral kinetics and clinical data were carried out. Data on viral loads, clinical laboratory results, ART regimen, and sociodemographic data were gathered. Viral loads analysis was undertaken using the COBAS AmpliPrep/COBAS TaqMan HIV-1 test, v2.0. Univariate and multivariate analyses were carried out to assess the variables related to virologic response.

**Results:**

Complete data was obtained for a total of 902 PLWH in this study. The average age was 45 ± 15.30 years, and 72.62% were female. The majority, 89.02% (835/902), had been on the DTG+3TC+TDF regimen. Over 60% had undetectable viral loads (<50 copies/mL). Univariate analysis shows a significant relationship between gender and virologic response, with females having a lower likelihood of virologic failure (OR: 0.60, 95% CI: 0.39–0.93, p-value = 0.024). In multivariate analysis, the duration of ART had various relationships with virologic response, with the odds ratio for two years reaching near significance (OR: 1.88, 95% CI: 0.98–3.59, p = 0.057). PLWH with viral loads >1000 copies/mL were 11.20% (101/902) while viral suppression, which was at detectable limits (>50 - ≤ 1000 cp/mL), was 13.08% (118/902) showing high rates of viral suppression.

**Conclusion:**

The presence of virologic failures was of concern despite the high rates of viral suppression that DTG-based ART demonstrated. Undetectable viral suppression was higher than detectable viral suppression. Regular monitoring of viral kinetics, adherence, and comorbidities is essential to meeting the United Nations program on HIV/AIDS (UNAIDS) 95-95-95 targets and providing efficient therapeutic approaches for PLWH.

## Introduction

The management of the human immunodeficiency virus (HIV) has seen tremendous improvement since the introduction of the antiretroviral therapy drug, dolutegravir (DTG) [1]. DTG-based regimens have shown better durability, efficacy, and tolerability compared to other regimens [2,3]. The majority of sub-Saharan African (SSA) countries have accepted and widely rolled out DTG-based antiretroviral therapy (ART) as first- and second-line treatment regimens as recommended by the World Health Organization (WHO) [2]. DTG-based ARTs have been shown to improve the quality of life of persons living with HIV (PLWH), most especially for those with high ART adherence. High genetic barrier to resistance, strong binding affinity to integrase, favourable pharmacokinetics, minimal drug-drug interactions, rapid viral suppression, efficacy in treatment-experienced patients, favourable safety profile, wide area of activity, synergistic effects in combination therapy, and positive impact on immune reconstitution define the efficacy and superiority of DTG-based therapy. All of these factors together help to explain its central importance in contemporary HIV treatment programs [3].

Since the introduction of DTG-based regimens as a first-line treatment option for PLWH in SSA countries like Ghana, there has been an increase in viral suppression; however, data continue to suggest that the UNAIDS agenda 95-95-95 is far from being met [4].

In recent years, available clinical data have consistently established that viral loads that are undetectable cannot be transmitted to another individual [5,6]. Therefore, to be able to achieve undetectable viral load levels, one must respond better to their ART regimen and adhere to the medication schedule. From the initiation or the switch from a previous ART regimen to a DTG-based regimen, it is imperative that the viral kinetics of PLWH be known as it determines HIV disease progression [7,8] and can inform assessment of the effectiveness of treatment and future decisions. Several studies have shown that despite the availability of ART for PLWH in Ghana, the quality of ART services rendered to such persons remains poor [9] which can partly be blamed for the country missing out on the previous UNAIDS agenda 90-90-90.

Understanding the viral kinetics will help in monitoring PLWH who are responding well to their medication, those developing drug resistance mutations (DRMs) [10,11] and those experiencing treatment failures for precise action and decisions in their treatment schedule. With much awareness of HIV disease progression [12] and efforts to therapeutic intervention, it is important to know the dynamics of viral kinetics of PLWH and when a steady state is achieved. Since the introduction of DTG-based regimens in Ghana, many PLWHs have had undetectable viral loads; however, viral load testing in developing countries like Ghana is very sporadic due to insufficient funding for PLWH. In view of this, there is little data on the viral regime of PLWH, hence the importance of studies like this to monitor the cross-section of the population for viral loads..

This study therefore used a retrospective and prospective approach to select patients attending HIV clinics in the Cape Coast metropolis to assess their viral load kinetics over the past three (3) years. This could help inform strategies aimed at increasing the numbers of PLHW who are virally suppressed and achieve undetectable viral load limits, as well as effective therapeutic strategies in ensuring the attainment of UNAIDS agenda 95-95-95 by the year 2030.

## Methods

### Study design and area

A retrospective analysis of clinical data and prospective analysis of viral loads among persons living with HIV on a DTG-based ART regimen were carried out at Ewim Polyclinic, Metropolitan Hospital, and Cape Coast Teaching Hospital HIV clinics within the Cape Coast metropolis in the Central Region of Ghana.

### Study population

**Fig 1:**
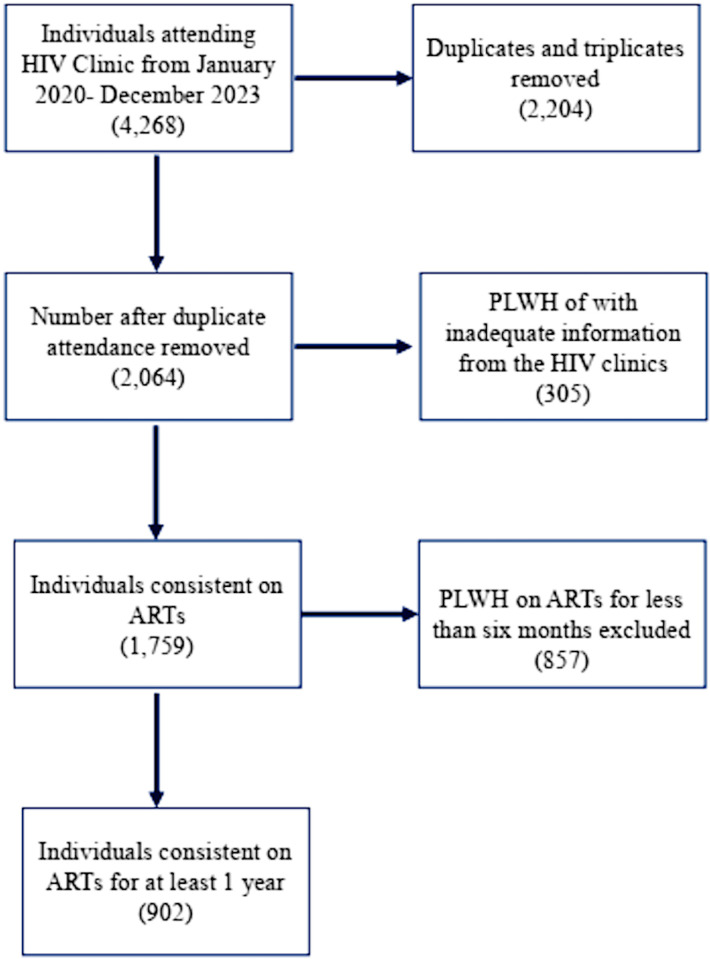
Flow chart showing the recruitment strategy of the study population of PLHW.

The retrospective arm of the study analyzed data from the Lightwave Health Information Management System (LHIMS) and used the available data to select PLWH that fit the inclusion criteria. Fig 1 represents a flowchart on the recruitment of the study participants. A total of 902 PLWH who had been on ART for at least six months and who agreed to provide samples for the study’s prospective arm from the biorepository at the Pharmacogenomics and Genomic Medicine Lab (https://pgmg-lab.com/). PLWH who have not been on ART for at least 6 months and pregnant women were excluded from the study.

### Data collection

Socio-demographic data of study participants were extracted from LHIMS and folders from 12^th^ April to 30^th^ May 2023. The data included age, gender, duration on ART, ART regimen, clinical laboratory data (RFT, LFTs, FBC, and viral loads), baseline, and previous viral loads for a minimum of two years. This data was used to follow up with PLWH in the clinic for blood sampling. Estimated Glomerular Filtration Rate was calculated according to the CKD-EPI formula [13]. The various stages of renal dysfunction were therefore classified based on the eGFR as follows:

- Stage 1 with normal or high GFR (GFR > 90 mL/min/1.73 m2)
- Stage 2 Mild CKD (GFR = 60–89 mL/min/1.73 m2)
- Stage 3A Moderate CKD (GFR = 45–59 mL/min/1.73 m2)
- Stage 3B Moderate CKD (GFR=30–44 mL/min/1.73 m2)
- Stage 4 Severe CKD (GFR = 15–29 mL/min/1.73 m2)
- Stage 5 End Stage CKD (GFR < 15 mL/min/1.73 m2)

### Blood sample collection

To determine viral load, 5 ml of blood were collected from each participant into EDTA vacutainer tubes from June 1 to December 22, 2023. Viral load was measured using the COBAS AmpliPrep/COBAS TaqMan HIV-1 test, v2.0 (Roche, Switzerland), following the manufacturer’s instructions. This assay has an analytical sensitivity of 20 HIV-1 RNA copies/mL and a specificity of 100% [14].

### Ethical approval

The Ethical Review Committee of the Cape Coast Teaching Hospital, Cape Coast, Ghana (CCTHERC/EC/2020/2020/109) authorized this study. Participants in the study provided verbal informed permission after receiving a thorough explanation of the process in the language and/or dialect that they best understood in the presence of a witness. Children who were included in the study had their parents/guardians’ consent for the study before their samples were taken.

### Quality assurance

All Standard Operating Procedures (SOPs) on the sample collection in this study were conducted in accordance with the International Conference on Harmonization of Good Clinical Practice and followed the principles of the Declaration of Helsinki.

### Statistical analysis

Data was entered and stored using Microsoft Excel, cleaned, and analyzed with STATA version 18 (STATA Corp., Texas, USA). A descriptive statistics of socio-demographic and other attribute data was performed using acceptable measures of central tendencies. Univariate and multivariate analyses were performed and presented, with p-values < 0.05 considered statistically significant. The models of multivariate analysis contained factors that were undertaken to evaluate the likely effect on viral load.

## Results

A total of 902 participants were included in this study. Table 1 presents their demographic and clinical characteristics. The mean (±SD) age of study participants was 45.06 ± 19.23 years, with 41% of participants falling between ages 45 and 60. The number of male participants was 27.38% (247/902), while 72.62% were females (Table 1). This represents a Male: Female ratio of approximately 1:3.

**Table 1:** Demographic characteristics and clinical data of study participants.

Characteristic	LPV/r-based (n = 8)	DTG-based (n = 835)	EFV-based (n = 56)	NVP-based (n = 3)
**Age, years (Mean ± SD)**	8.00 ± 2.61	45.06 ± 15.30	47.13 ± 13.40	24.50 ± 16.26
**Age groups, years**	N (%)	N (%)	N (%)	N (%)
0-9	4 (50)	24 (2.87)	0	0
10-14.	2 (25)	25 (2.99)	1 (1.79)	1 (33.33)
15-24	0	36 (4.31)	2 (3.57)	0
25-44	0	229 (27.43)	21 (37.50)	1 (33.33)
45-60	0	339 (40.60)	24 (42.86)	0
61+	0	99 (11.85)	8 (14.29)	0
Not known	2 (25)	83 (9.90)	0	1 (33.33)
**Gender**				
Male	5 (62.50)	219 (26.23)	22 (39.29)	1 (33.33)
Female	3 (37.50)	616 (73.77)	34 (60.71)	2 (66.67)
**Minimum number of years on ART**				
1	1 (12.50)	234 (28.02)	0	0
2	0	101 (12.10)	4 (7.14)	0
3	0	46 (5.51)	1 (1.79)	0
4	0	22 (2.63)	1 (1.79)	0
5	0	30 (3.59)	3 (5.36)	0
≥ 6	0	43 (5.15)	0	1 (33.33)
Not known	8 (87.50)	359 (42.99)	47 (83.93)	2 (66.67)
**Most recent viral load measurement (copies/mL)**				
<50	5 (62.50)	516 (61.80)	16 (28.57)	1 (33.33)
51-999	0	114 (13.65)	4 (7.14)	0
>1000	1 (12.50)	96 (11.50)	1 (1.79)	1 (33.33)

PLWH were on 4 different ART regimens, with 93% of them on a DTG-based regimen. These were efavirenz-based (AZT+3TC+EFV and EFV+3TC+TDF), nevirapine-based (NVP+3TC+AZT), lopinavir/ritonavir-based (LPV/r+3TC+AZT and LPV/r+3TC+ABC), and dolutegravir-based (DTG+3TC+ABC, DTG, DTG+3TC+AZT, and DTG+3TC+TDF). Sixty percent of (60%) PLWH had achieved undetectable viral load limits (<50 cp/mL).

Most participants (803/902) used the 3-in1 ART combination regimen of dolutegravir, lamivudine and tenofovir (DTG+3TC+TDF). The single regimen DTG was administered to only one study participant (0.11%) (Table 2).

**Table 2:** ART regimen administered to PLWH at the HIV clinics at Cape Coast Metropolitan, Ghana (N = 902).

ART Dispensed	Number, N	Frequency, %
AZT+3TC+EFV	2	0.22
NVP+3TC+AZT	2	0.22
DTG+3TC+ABC	19	2.11
DTG	1	0.11
DTG+3TC+AZT	12	1.33
DTG+3TC+TDF	803	89.02
LPV/r+3TC+AZT	3	0.33
LPV/r+3TC+ABC	5	0.55
EFV+3TC+TDF	54	5.99
Not included	1	0.11

Liver function parameters are categorised by ART regimen in Table 3. Due to resource constraints, we present data for approximately 50% of the study population. Of the 454 individuals, 76.16% had normal ALT levels and 63.0% had normal AST levels following a DTG-based regimen.

**Table 3:** AST and ALT levels/ activities according to ART Regimen.

Parameters	ART Regimen Backbone				
	**DTG-based** **n (%)**	**EFV- based** **n (%)**	**Lopinavir-based** **n (%)**	**NVP-based** **n (%)**	**p-value**
**AST (N = 454)**					
Below normal (<5 IU/L)	2 (0.4)	0	0	0	0.2029
Normal (5–34)	286 (63.0)	9 (2.0)	1 (0.22)	0	
Above normal (>34)	145 (31.94)	5 (1.10)	5 (1.10)	1 (0.22)	
**ALT (453)**					0.7731
Below normal (<10)	60 (13.25)	2 (0.44)	2 (0.44)	0	
Normal (10–50)	345 (76.16)	12 (.65)	4 (0.88)	1 (0.22)	
Above normal (>50)	27 (5.70)	0	0	0	

Haematocrit was normal in 87.31% of DTG users, platelet count varied significantly across regimens (p < 0.001), and other blood parameters did not show significant differences (p > 0.05). Of the 520 participants, approximately 51% of PLWH prescribed on DTG-based regimen had normal haemoglobin levels, while 38.27% had mild anaemia (Table 4).

**Table 4:** Selected Haematological Indices according to the ART Regimen.

Parameters	ART Regimen Backbone				
**DTG-based** **n (%)**	**EFV- based** **n (%)**	**Lopinavir-based** **n (%)**	**NVP-based** **n (%)**	**p-value**
**Hemoglobin Count (N = 520)**					**0.016**
Severe anemia (<8g/dL)	28 (5.38)	2 (0.38)	0	0	
Mild anemia (8-11g/dL)	199 (38.27)	9 (1.73)	7 (1.35)	2 (0.38)	
Normal hemoglobin levels (≥12g/dL)	267 (51.35)	6 (1.15)	0	0	
**Hematocrit (N = 520)**					
below normal	33 (6.35)	4 (0.77)	1 (0.19)	1 (0.19)	**0.048**
Normal	454 (87.31)	13 (2.5)	6 (1.15)	1 (0.19)	
above normal	7 (1.35)	0	0	0	
**Red Blood Cells (N = 519)**					0.973
Below normal (<=2.4)	10 (1.93)	0	0	0	
Normal (2.4–5.5)	461 (88.82)	17 (3.28)	7 (1.35)	2 (0.39)	
Above normal (>5.5)	22 (4.24)	0	0	0	
Total	493	17	7	2	
**White Blood Cells (N = 520)**					0.837
Below normal (<3x10^3^/µL)	125 (24.04)	2 (0.38)	1 (0.19)	0	
Normal (3-17x10^3^/µL)	368 (70.80)	15 (3.00)	6 (1.15)	2 (0.38)	
Above normal (17x10^3^/µL)	1 (0.19)	0	0	0	
**Platelet (N = 519)**					**0.000**
Below normal (<50x10^3^/µL)	14 (3.0)	0	1 (0.19)	0	
Normal (50–400 x10^3^/µL)	465 (90.0)	17 (3.28)	4 (0.77)	1 (0.19)	
Above normal (>400 x10^3^/µL)	14 (3.0)	0	2 (0.39)	1 (0.19)	

Renal function parameters by ART regimen are shown in Table 5. Of the 460 participants, 35% of DTG users had normal blood urea, 76.52% of DTG-based regimen users had normal creatinine levels, and 11.74% had above-normal levels. eGFR varied significantly between regimens (p = 0.0063), with 41.14% of DTG users having moderately reduced kidney function.

**Table 5:** Renal function according to the ART Regimen.

Parameters	ART Regimen Backbone				
	DTG-based n (%)	EFV- based n (%)	Lopinavir-based n (%)	NVP-based n (%)	p-value
**Creatinine (N = 460)**					**0.000**
Below normal (<53)	32 (7.0)	2 (0.43)	4 (1.0)	1 (0.22)	
Normal (53–123.8)	352 (76.52)	12 (2.61)	2 (0.43)	0	
Above normal (>123.8)	54 (11.74)	1 (0.22)	0	0	
**Blood Urea (N = 1,015)**					0.506
Below normal (<2.14)	619 (61.0)	2 (0.20)	2 (0.20)	0	
Normal (2.14–7.12)	355 (35.0)	13 (1.28)	3 (0.30)	1 (0.10)	
Above normal (>7.12)	19 (2.0)	0	1 (0.10)	0	
**eGFR (N = 316)**					**0.0063**
G5-End Stage Kidney Failure (ESKF) (<15)	6 (2)	0	0	0	
G4-Severely Reduced GFR (15–29.99)	4 (1)	0		0	
G3b-Moderate to severely reduced GFR (30–44.99)	41 (13)	1 (0.3)	0	0	
G3a-Moderately Reduced GFR (Frank CKD)	85 (27)	2 (0.6)	0	0	
G2-Mildly Reduced GFR (60–89.99)	100 (32)	5 (1.6)	0	0	
G1-Kidney damage with Normal or Increased GFR (90–120)	62 (20)	4 (1.2)	5 (1.6)	1 (0.3)	

**Fig 2:**
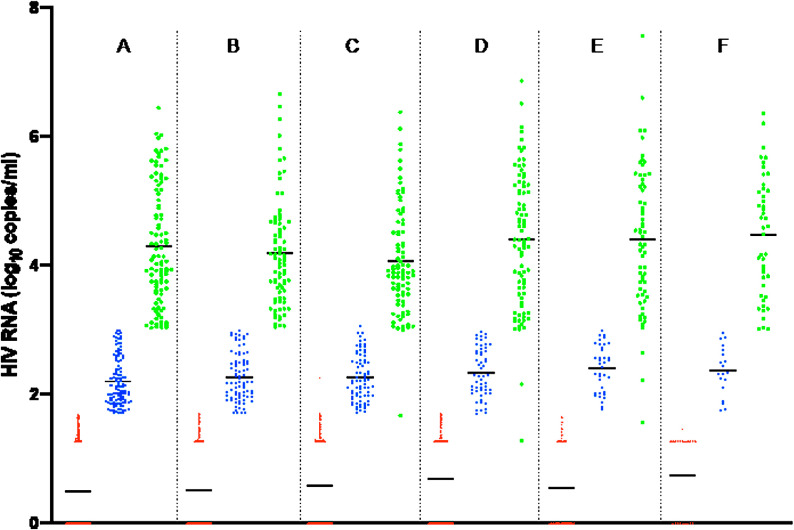
Viral load measurements over 30 months for PLWH on DTG-based antiretroviral therapy. A-F represents the number of viral load measurements with A representing the most recent viral load and F representing the initial viral load measurement. **Legend: Red-undetectable viral load (<50cp/ml); blue- virally suppressed (51-1000cp/ml); green- virologic failure (>1000cp/ml).**

The 3-year viral load regime at 6-month intervals showed that most of the PLWHs had undetectable limits of viral load (<50 cp/mL). There was a strong virologic response (VR) to the DTG-based therapy across the 30-month period (Fig 2). The number of PLWH who had viral loads >1000 cp/mL decreased over the 2–3-year period from June 2021 to December 2023 (F to A), with 60% achieving viral loads of <50 cp/mL and 13% achieving viral suppression by the most recent viral load measurement (A in Fig 2). A significant number of participants (n = 185) had viral load measures with target not detected (red dots on x-axis) (<7 cp/mL).

**Fig 3:**
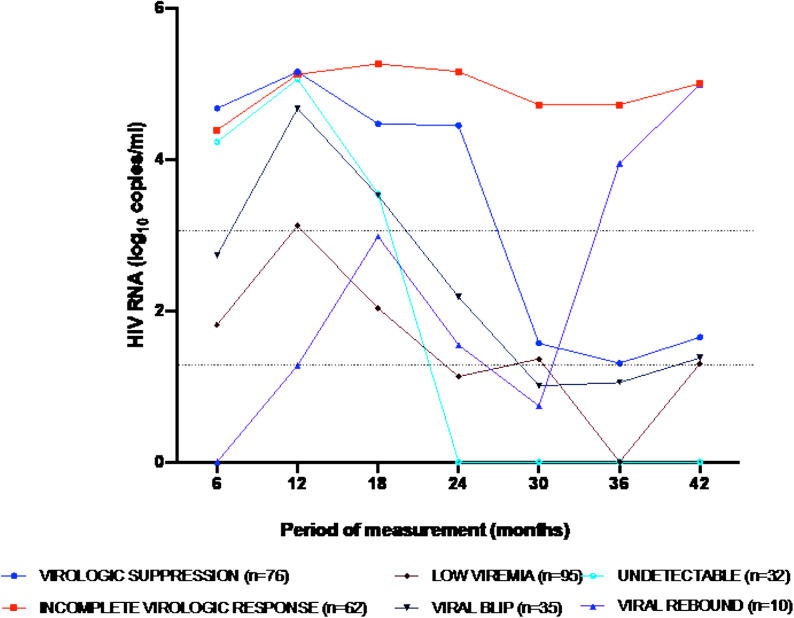
HIV viral load of selected patients showing different phases of viral load kinetics. The various phases and points are average viral load for the number of selected patients. The first line across from the origin on the y (viral load)-axis represents viral loads of 50cp/mL and the second line represents viral loads of 1000cp/mL.

Viral load data was obtained for a selected 310 PLWH over the past 3.5-years period at 6-month intervals, to elucidate on viral kinetics (Fig 3). The viral load data were plotted over periods of measurement. Sixty-two (62) PLWH had incomplete virologic response, with viral load above 1000cp/mL since diagnosis. There were PLWH who showed viral rebound with viral loads of >1000cp/mL for the past 1-year after having achieved viral suppression (<1000cp/mL) previously. This data shows that there could be a challenge with adherence for such PLWH who could have reneged on their therapy since they thought they were ‘cured’.

Univariate analysis shows a significant relationship between gender and virologic response, with females having a lower likelihood (OR: 0.60, 95% CI: 0.39–0.93, p-value = 0.024) (Table 6). However, in multivariate models (crude and Model 1), the relationship becomes non-significant, suggesting that gender may not be an independent predictor of virologic response. Age of participants showed some relationships approach significance; however, many are not statistically significant, showing the complex link between age and the chance of a positive virologic response. The duration of ART had various relationships with virologic response, with the odds ratio for two years reaching near significance (OR: 1.88, 95% CI: 0.98–3.59, p-value = 0.057). This shows that the length of ART usage may affect the likelihood of a virologic response.

**Table 6:** Univariate and multivariate analysis of factors associated with virologic response of study participants.

	Univariate		Multivariate (crude model)	
	OR (95% CI)	p-value	OR (95% CI)	p-value
**GENDER**				
Male	1		1	
Female	0.60 (0.39-0.93)	**0.024**	1.41 (0.26-7.46)	0.573
**AGE, years**				
0-9	1		1	
10-14	2.00 (0.42- 9.42)	0.380	5.64 (0.13- 243.19)	0.259
15-24	2.21 (0.53- 9.25	0.279	1	–
25-44	1.05 (0.29- 3.75	0.941	0.89 (.01- 54.07)	0.840
45-60	0.90 (0.26-3.16)	0.871	5.97 (0.15- 240.48)	0.398
61+	1.47(0.39- 5.56)	0.567	6.51 (.14- 293.12)	0.375
**ART REGIMEN DISPENSED**				
AZT+3TC+EFV	1	–		
DTG+3TC+ABC	6.30 (0.63-63.64)	0.117		
DTG+3TC+AZT	9.50 (0.83-109.23)	0.071		
DTG+3TC+TDF	2.80 (0.37-21.16)	0.319		
LPV/r+3TC+AZT	19.00 (0.62- 583.38)	0.092		
**MINIMUM YEARS ON ART**				
1	1			
2	1.88 (0.98- 3.59)	0.057		
3	0.83 (0.27- 2.53	0.748		
4	1.32 (0.36- 4.83)	0.671		
5	0.83 (0.24- 2.94)	0.777		
6 and above	0.38 (0.09- 1.64)	0.193		
**ART Regimen Back bone**				
Lopinavir-based	1		1	
DTG-based	0.77 (0.09- 6.66)	0.812	0.06 (0.0005-6.42)	0.530
EFV-based	0.25 (0.01- 4.73)	0.355	**–**	**–**
NPV-based	5 (0.15- 166.59	0.368	**–**	**–**
**Viral loads (cp/ml)**				
<50	1		1	
51-1000	2.08 (1.05-4.13)	**0.037**	0.93 (0.08-11.24)	0.955
>1000	3.21 (1.69-6.18)	**0.000**	7.93 (1.52-41.57)	**0.014**

Model 1: adjusted for ART backbone and viral load before most recent viral load.

The viral load can contribute to virologic response and this is shown in a strong positive relationship with the virologic response (OR: 3.21, 95% CI: 1.69–6.18, p-value = 0.000), emphasizing the significant role of viral load control in determining the likelihood of viral suppression (Table 3).

## Discussion

The quality of HIV care is very important to achieving the UNAIDS agenda 95-95-95 [15]. As a measure of achieving the 3rd part of the agenda, which is 95% of individuals on ART having achieved viral suppression, it is important that the viral kinetics of PLWH are monitored so that if there is any change in the viral loads, efforts will be made in time to correct it. In this study, therefore, efforts were made to study the viral kinetics of PLWH at 6-month intervals to monitor if there is any change in viral load profile over time. We undertook a retrospective and prospective study where we leveraged electronic health records (EHRs) at HIV clinics within the Cape Coast Metropolis to evaluate the viral kinetics of PLWH over a 6-month period for a minimum of 30 months. The ratio of Male: Female was 1:3 owing to the fact that some infected males unknowingly infect females before their diagnosis, which is consistent with published data that shows the vulnerability of females to HIV infection compared to males [16,17]. We observed PLWH were given ARTs with LPV/r-based, DTG-based, EFV-based, and NVP-based backbone medications over the period, with the majority of PLWH being on DTG-based ART and the least being on an NVP-based regimen. The study population revealed that PLWH on the DTG-based regimen had an average age of 45 years, with a larger proportion being females, many of whom had been on the medication for more than 6 years showing that most of them had the disease before the age of 40. This is consistent with global trends, which show that females make up a larger proportion of PLWH [1,18–21]. In a previous study in Hong Kong, it was observed that the average age of PLWH who are prescribed with LPV/r-based regimen was highly consistent with our study with the average age of 8 years [22]. Key markers of liver function, AST (aspartate amino-transferase) and ALT (alanine amino-transferase) levels in HIV patients must be closely monitored. Regular assessment of AST and ALT levels is essential as HIV infection as well as ARTs used to treat it may greatly impair liver function. Early indicators of liver damage should be sought for. Increased levels of these enzymes might point to liver inflammation or damage brought on by HIV itself, co-infections include Hepatitis B or C, or ART-related hepatotoxicity [3]. Since the liver is essential for the metabolising of ARTs, liver malfunction may potentially affect drug safety and effectiveness. Monitoring AST and ALT levels thus helps identify liver-related issues early on, allowing quick changes to medication and thus improving general patient care and prognosis [7]. Table 3 shows that most of patients on dolutegravir (DTG)-based regimens had normal AST and ALT values, however, a considerable percentage (31.94%) had high AST, which would point to either minor hepatic damage linked with ART or hepatocellular stress similar to a study in Nigeria by Anyanwu et al [23]. By contrast, regimens based on efavirenz (EFV), lopinavir (NVP), and nevirapine (NVP) revealed much less percentages of patients with aberrant AST and ALT values. These could also be accounted for by the smaller numbers of PLWH who were on these ARTs. Although statistically AST and ALT, were not significant, the greater prevalence of aberrant liver function indicators in DTG-based regimens calls further research on possible hepatotoxic consequences.

Counts of red blood cells (RBC) did not indicate any significant change across regimens (p = 0.973) just as presented by Echefu et al in 2023. With no significant change across regimens (p = 0.837), white blood cell (WBC) levels were mostly within normal limits, contrary to their study, total WBC count appreciated with those who used DTG regimen compared with EFV and ritonavir regimens [24]. Platelet counts differed significantly (p = 0.000), however, DTG-based regimens displayed both greater and lower extremes than the other ART treatments showing signs of increased platelets as similar to a recent study [25]. These results draw attention to the possible haematological effects of ART, especially DTG-based regimens, which can call for more frequent and strict monitoring of anaemia and platelet abnormalities.

There was an observed significant difference in creatinine levels (p = 0.000)_and eGFR (p = 0.0063) categorized by ART use, especially DTG. This is similar to a study conducted in Zambia in 2023 where some individuals had severe renal impairment after long-term use of DTG [26]. Participants on DTG-based regimens (11.74%) showed elevated creatinine levels in comparison to others, indicating a possible effect on renal function. By contrast, regimens based on EFV, LPV, and NVP showed less cases of creatinine increase. Although blood urea levels did not vary much across regimens (p = 0.506),

These results imply that, in comparison to EFV, NVP, or lopinavir-based regimens, DTG would have a greater risk of renal problems. Although serious kidney dysfunction was relatively rare, the greater frequency of increased creatinine and lower eGFR in DTG users calls for vigilant observation especially in individuals with pre-existing renal risk factors including diabetes or hypertension [27].

Whether these effects are dose-dependent or altered by concurrently used medications should be investigated further.

The majority of PLWH were on the DTG+3TC+TDF regimen, demonstrating its importance in the therapy landscape. However, there were still PLWH who were on EFV/3TC/TDF, as these PLWH had previously shown adverse drug events (ADEs) to the new DTG regimen and therefore were still on the old regimen. Despite its effectiveness, DTG-based ART has been implicated in ADEs [28,29] although there is a paucity of data in Ghana.

Since the DTG-based ART was the most prescribed medication, the viral load profile of PLWH, who had been on for a minimum of 30 months, was observed. This shows that the number of PLWH who have achieved viral suppression (<1000 cp/ml) and undetectable levels (<50 cp/ml) over the period has increased, likewise those who had virologic failure (>1000 cp/ml). Dolutegravir (DTG)-based antiretroviral therapy (ART) regimens demonstrate high efficacy due to DTG’s role as an integrase strand transfer inhibitor, which obstructs the integration of viral DNA into the host genome—a pivotal phase in HIV replication—resulting in rapid and sustained viral suppression, with over 80% of PLWH attaining undetectable viral loads in studies involving diverse populations [30,31].

We further analyzed specific individual data according to whether they have achieved virologic failure or viral suppression, just as has been seen in several studies in Africa [5,19,32,33]. We observed some persons had consistently not achieved viral suppression >1000 cp/mL over the 30-month minimum period. In a study undertaken recently, it was shown that 10.8% are in virologic failure due to non-adherence of ART [18]. Non-adherence is a problem in PLWH in Ghana, as several studies [33–36] have shown the lack of consistency of PLWH in taking their medications. So, for the PLWH involved in this study, it could either be as a result of non-adherence or treatment failure. This is of concern to achieving agenda 95-95-95 as approximately 15% of the PLWH had virologic failure.

It is known that several factors influence the viral load outcome in PLWH. We therefore undertook a virologic response univariate and multivariate model analysis for association with factors that could influence virologic response in our cohort. There was significant association (OR = 0.60, 95% CI: 0.39–0.93, p = 0.024) between being a female and having a lower viral load in the univariate model. Similarly, it has been shown in previous studies that females have lower viral loads than males because they are more likely to adhere to their medications than males and respond better to treatment [37–39].

A limitation of this study was that there was missing, incomplete, or no data from the electronic health records system for some of the patients, such as WHO staging, blood pressure, weight, height, and age. This made it difficult for us to involve all the patients in our analysis, although we obtained enough cohorts to undertake the study. Due to limited funding, the clinical assays (FBC, LFT, RFT) were done for nearly half of the population and repeats were not possible.

## Conclusion

Viral kinetics among PLWH on dolutegravir-based antiretroviral therapy provided useful insights into treatment response, factors affecting viral suppression, and the need for connected monitoring and management measures. Viral suppression was high in PLWH on the DTG-based regimen; however, virologic failures were observed for some PLWH in this study, which is a concern.

## Abbreviations

DTG: Dolutegravir
3TC: Lamivudine
ABC: Abacavir
AZT: Zidovudine
TDF: Tenofovir disoproxil
WHO: World Health Organization
EFV: Efavirenz
LPV/r: Lopinavir/ritonavir
NVP: Nevirapine
UNAIDS: United Nations Programme on HIV/AIDS
